# Functional Outcome after Treatment of Aggressive Tumours in the Distal Radius: Comparison between Reconstruction using Proximal Fibular Graft and Wrist Fusion

**DOI:** 10.5704/MOJ.1811.004

**Published:** 2018-11

**Authors:** CY Choo, AM Mat-Saad, WS Wan-Azman, Z Wan, MZ Nor-Azman, S Yahaya, WI Faisham

**Affiliations:** Department of Orthopaedics, Hospital Universiti Sains Malaysia, Kubang Kerian, Malaysia; *Plastic Surgery and Reconstructive Science Unit, Hospital Universiti Sains Malaysia, Kubang Kerian, Malaysia; **Department of Orthopaedics, Prince Court Medical Centre, Kuala Lumpur, Malaysia

**Keywords:** distal radius tumour, reconstruction, fusion, outcome

## Abstract

**Introduction:** Restoration of a functional hand is the ultimate goal following a distal radius tumour resection. The early outcomes of mobile wrist reconstruction are satisfactory; however, long-term results are unpredictable due to late wrist instability and degenerative arthritis. Our aim is to compare mobile wrist reconstruction with wrist fusion (pan-carpal fusion) in our cohort of patients.

**Materials and Methods:** A retrospective cohort study was performed for functional outcomes of all patients who underwent resection for distal radius tumour and treated with either fusion or reconstruction of the wrist in a single institution from years 2000-2013 with a minimum of three years follow-up.

**Results:** Eleven patients were included in the study, six of whom had wrist reconstruction with proximal fibula graft and the remaining five wrist fusion, with a mean follow-up of 6.3 years. The mean Musculoskeletal Tumour Society (MSTS) score was 82.78%, ranging from 70% to 93.3%. Average grip strength compared to the normal contralateral hand was 60.0% for total wrist fusion, which was better than wrist reconstruction with 58.07%. There was no difference in the functional outcome between fusion and mobile reconstruction in our study. Osteoarthritis changes and subluxation of the wrist joint were the most common findings in the long-term follow-up for this group.

**Conclusion:** There was no difference in the functional outcome of the long-term follow-up between the two groups.

## Introduction

Surgical resection of the distal radius tumour creates a massive bony defect and warrants reconstruction in order to achieve painless hand function. Few options, including resection arthroplasty, the use of a non-vascularised or vascularised autogenous fibular graft and allograft replacement, custom prosthetic replacement and ulnar translocation have been used for the reconstruction of this bone defect^1-11^. Proximal fibular graft has a near anatomical similarity in shape and size to the distal radius and is suitable for mobile reconstruction. The early functional results were satisfying, but late instability and degenerative changes of the carpo-fibular joint are frequently observed due to the relative incongruence of the carpo-fibular articular surfaces^10^.

The aim of this study was to compare the functional outcome between total wrist fusion (pan-carpal fusion) and mobile wrist reconstruction with fibular grafting after wide excision of the distal radius tumour.

## Materials and Methods

This is a retrospective cohort study of long-term function and hand grip strength in all patients who underwent distal radius resection for primary tumour from January 2000 to December 2013 with a minimum follow-up period of three years. Routine radiographs were taken at three monthly intervals for one year and, thereafter, every six months to evaluate radiographic union. Radiographs were taken during the final evaluation to detect wrist subluxation and arthritis.

The functional outcome was evaluated by a single researcher using the Musculoskeletal Tumour Society scoring system (MSTS)^11^, which comprises six components rated on a five-point Likert-type scale with 0 being the worst score and 5 as the normal full function. The components were: pain, function, emotional acceptance, hand positioning, manual dexterity and lifting ability. Handgrip strength was assessed in comparison with the opposite normal hand using Jamar hand dynamometer. All patients agreed for interview, and functional outcomes were included. The conversion of reconstruction surgery to total wrist fusion in the arthrodesis group was considered later. Complications of the procedure, either on the donor or recipient side, or further surgery (if performed) were recorded. The mean data were evaluated for comparison of function and grip strength using SPSS version 20.0. The study was approved by the Human Research Ethics Committee, Universiti Sains Malaysia (USM/JEPeM/14090314).

## Results

**Fig. 1:**
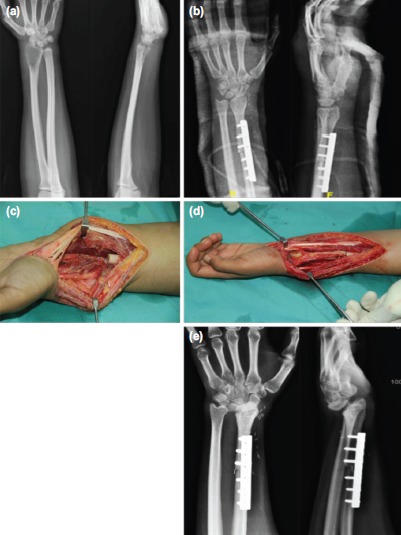
Mobile wrist reconstruction in a 30 year-old lady teacher with left distal radius giant cell tumour. (a) Pre-operative radiograph. (b) Early post-operative radiograph showing good reconstruction. (c) Intra-operative photograph showing bony defect post-wide resection of left distal radius tumour. (d) Wrist reconstruction with non-vascularised proximal fibular graft. (e) Left wrist subluxation noted three years after surgery with arthritic changes, but daily activities were unaffected.

**Fig. 2:**
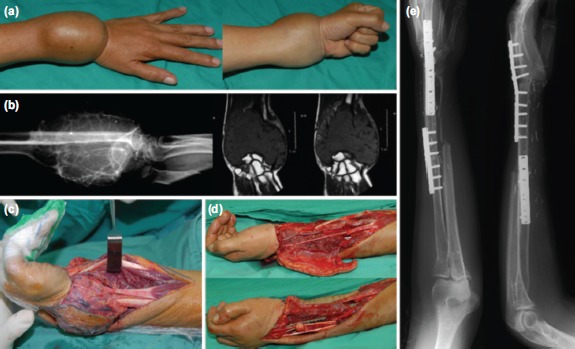
Wrist fusion in a 42 year-old man with long standing swelling for five years. (a) Pre-operative photo. (b) Pre-operative radiograph and MRI revealed expansile septated lesion involving the entire distal radius with distal extension to proximal row of carpus and distal ulna. (c) Resection of radius and ulna with proximal row carpus. (d) Intra-operative photo showing the defect post-resection (top) and reconstruction with vascularised fibular graft (bottom). (e) Plain radiograph taken eight years post-operatively with stable wrist fusion with good hand grip.

A total of eleven patients were included in this study. There were four males and seven female patients with the mean age of 36.6 years (range: 19-56 years of age). The mean duration for the follow-up was 6.3 years (range: 4-14 years). Surgery involved the dominant hand for five patients. There were nine patients with Campanacci grade III giant cell tumour, one patient each with osteosarcoma and aneurysmal bone cyst^2^. Six patients underwent wrist reconstruction procedures, and five patients underwent total wrist fusion (Table I). The decision of choosing the type of operation was based on the extent of local tumour extension and the patient’s occupational demands. Patients employed as manual workers and requiring a stable wrist and strong hand grip were counselled for wrist fusion (Fig. 1); whereas, for those with less demanding physical tasks that require more wrist movement or fine motor skills, wrist reconstruction was offered (Fig. 2). Three cases had undergone vascularised fibular graft that required soft tissue reconstruction and more extensive bony resection. Two patients had proximal row carpectomy for tumour clearance and all cases of vascularised fibular graft had fusion of the wrist. The mean resection length for the reconstruction group was 6.1cm compared to the fusion group of 11.6cm.

**Table I:** Comparison between wrist fusion and reconstruction of the distal radius tumour

Character	Reconstruction (6)	Fusion (5) (3 vfg*)	Statistic
Age (mean years)	36.5	36.6	
Gender			
Male	1	3	
Female	5	2	
Side of surgery			
Right	2	2	
Left	4	3	
Diagnosis			
GCT^†^	5	4	
ABC^‡^	1		
Osteosarcoma		1	
Functional outcome			Mann Whitney test (p-value)
Overall MSTS	25 (82.8%)	23(78%)	0.270
Pain	4	5	0.032
Function	4.5	3	0.153
Emotional acceptance	4	4	0.95
Hand positioning	5	5	0.95
Manual dexterity	5	4	0.077
Lifting ability	3	3	0.361
Grip strength (%)	58.1	62.51	0.580
Union time (weeks)	18.4 (1)	18.7 (2)	

* Vfg = vascularised osteo-fasciocutaneous fibula flap

† GCT = giant cell tumour

‡ ABC = aneurysmal bone cyst

The overall Musculoskeletal Tumour Society (MSTS) score ranged from 70 to 93.3%, with four ‘good’ results and seven ‘excellent’ results. The mean MSTS score for the wrist reconstruction group was 82.78%, and the total wrist fusion group was 78.0%. There was no statistically significant difference between the two groups overall functional outcomes (Mann-Whitney test, p=0.270). Evaluation of the pain component of MSTS revealed that the wrist reconstruction had a mean of 4.2 compared to 5.0 for total wrist fusion and was statistically significant (Mann-Whitney test, p=0.032). No patient with wrist fusion complained of pain on final evaluation. Average grip strength, when compared to the normal contralateral hand, was 62.51% for total wrist fusion which was slightly better than wrist reconstruction, 58.07%, but not statistically significant.

Radio-fibular non-union occurred in three cases. The union rate for wrist reconstruction was 83.3%; whereas, for total wrist fusion, it was 60%. The average time for union was 18.4 weeks for wrist reconstruction and 18.7 weeks for total wrist fusion. The non-union cases were treated with iliac bone grafting and all had eventually achieved union.

## Discussion

Mobile wrist reconstruction in distal radius tumour excision produced early and good functional outcomes; however, no long-term outcomes have been evaluated^1, 4-10^. There was no difference in the functional outcome between fusion and mobile reconstruction in our study. However, detailed analysis revealed that the pain component was significantly higher in the reconstruction group. Osteoarthritis changes and subluxation of the wrist joint were the most common findings in the long-term follow-ups for this group^2-4^. Three of our patients who underwent wrist reconstruction developed a subluxated wrist with pain. Carpal subluxation is commonly reported in the literatures and can occasionally be a disabling problem. Degenerative arthritis between the head of the fibula and carpal bones occurs due to articular incongruity and no articular remodelling; particularly in adults^2, 7.^

Arthrodesis produces a painless and stable wrist, though absence of motion, with minimal disability. The grip strength for the wrist fusion group was stronger compared to the reconstruction group. A stable and painless wrist is attributed to better tendon excursion and muscle strength for the grip^5, 8^. The vascularised fibular graft is performed if the tumour breached the cortex with soft tissue extension or destroyed the proximal carpus. Osteocutaneous vascularised fibular graft provides a good soft tissue cover for better tendon gliding and stability of wrist fusion for early rehabilitation. It was observed that the final outcome following massive resection, including proximal row carpectomy managed with wrist fusion and osteocutaneous vascularised fibular graft, produced good results with strong grip strength compared to wrist reconstruction. Arthrodesis of the wrist also produced good results as loss of wrist movement can be compensated by other joints and rotation of the forearm in daily life activities.

Recent literature has shown that there is no difference in long-term functional outcome between fusion and reconstruction group. Translocations of ulna, wrist arthrodesis using segmental iliac crest graft and complex procedure of segmental double barrel ulnar graft arthrodesis with Sauve-Kapandji procedure also produce equally good long-term outcome^12-14^.

One patient in the total wrist fusion group had pathological fracture which was treated with plate and screws. There was no major donor site morbidity; however, weakness of the extensor hallucis longus in the donor limb did occur.

## Conclusion

Wrist fusion had less chronic pain and better grip strength compared to reconstruction after distal radius tumour resection. However, there was no difference in the functional outcome in the long-term follow-up between the two groups.

## Conflict of Interest

The authors declare no conflicts of interest.
